# Sequential treatment for a patient with hemifacial microsomia: 10 year-long term follow up

**DOI:** 10.1186/s40902-015-0004-6

**Published:** 2015-02-05

**Authors:** Jeong-Seok Seo, Young-Chea Roh, Jae-Min Song, Won-Wook Song, Hwa-Sik Seong, Si-Yeob Kim, Dae-Seok Hwang, Uk-Kyu Kim

**Affiliations:** Department of Oral and Maxillofacial Surgery, School of Dentistry, Pusan National University, 20 Geumo-ro, Mulgeum-eup, Yangsan, 626-787 Korea

**Keywords:** Hemifacial microsomia, Distraction osteogenesis, Facial augmentation

## Abstract

Hemifacial microsomia (HFM) is the most common craniofacial anomaly after cleft lip and cleft palate; this deformity primarily involves the facial skeleton and ear, with either underdevelopment or absence of both components. In patients with HFM, the management of the asymmetries requires a series of treatment phases that focus on their interception and correction, such as distraction osteogenesis or functional appliance treatment during growth and presurgical orthodontic treatment followed by mandibular and maxillary surgery. Satisfactory results were obtained in a 9-year-old girl with HFM who was treated with distraction osteogenesis. At the age of 19, genioplasty and mandible body augmentation with a porous polyethylene implant (PPE, Medpor®, Porex) was sequentially performed for the functional and esthetic reconstruction of the face. We report a case of HFM with a review of the literature.

## Background

Hemifacial microsomia (HFM) is a deformity derived from the first and second branchial arches and primarily involves the facial skeleton and ear, resulting in the underdevelopment or absence of both components. HFM is the most common craniofacial anomaly in humans after cleft lip and cleft palate. A classification of HFM has been described by Pruzansky [1] and developed to qualify and quantify the severity of the deformity of the facial skeleton and associated soft tissues [2–6].

It is difficult to reconstruct hard and soft tissues of the deformed face in HFM, and the treatment of this condition requires multiple steps over a period of several years [7]. Costochondral grafts have been widely used for the reconstruction of deficient mandibular ascending ramus in children with Pruzansky–Kaban types IIB and type III HFM between the mid-1970s and mid-1990s [8]. Children with types I and IIA HFM were not treated until the end of pubertal growth [4]. In some instances, they were treated with functional orthodontic therapy to induce harmonious maxillomandibular growth [9]. Distraction osteogenesis (DO) was first performed in 1992 by McCarthy, in a patient with HFM [10]. We present the case of a 9-year-old girl who was diagnosed with HFM, and was treated with sequential distraction osteogenesis, genioplasty, and mandible body augmentation. She was later treated with a porous polyethylene implant (PPE, Medpor®, Porex) at 19 years of age for the functional and esthetic reconstruction of her face.

## Case presentation

**Figure 1:**
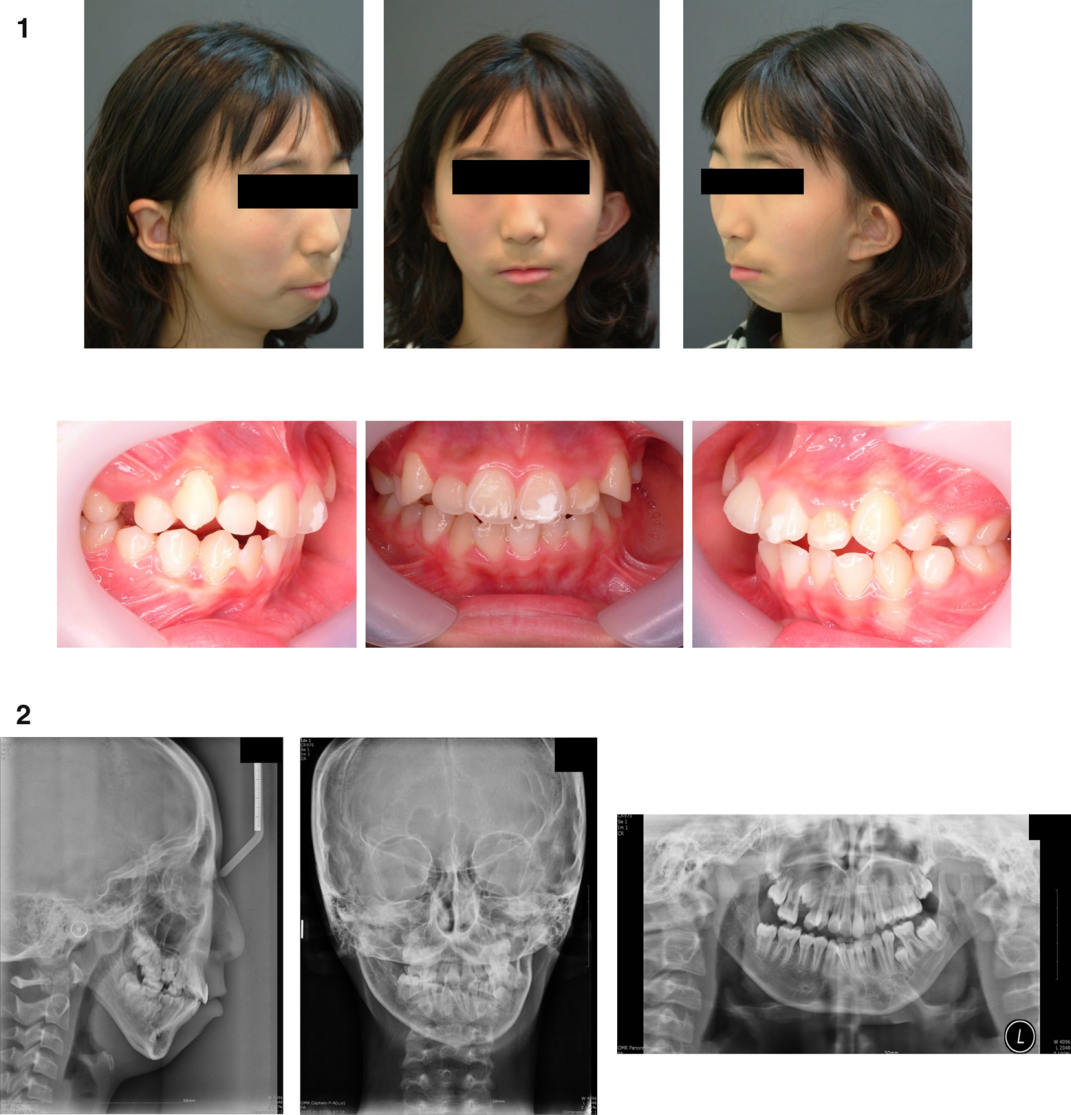
**Pre-operative view of patient.** **1.** Pre-operative extraoral and intraoral photographs. **2.** Pre-operative Skull lateral and AP views, Panoramic view.

A 9-year-old girl was referred to the Department of Oral and Maxillofacial Surgery, Pusan National University Hospital, Pusan, Republic of Korea, for the evaluation and treatment of a facial deformity. Clinical findings were as follows: micrognathia, lower incisor midline deviation towards the left side, hypoplasia of the mandibular condyle and coronoid process, deficiency in ramus height and length of the left mandibular body, and canting of the occlusal plane (Figure 1). Clinical and radiographic examinations indicated that she had HFM anomaly.

**Figure 2:**
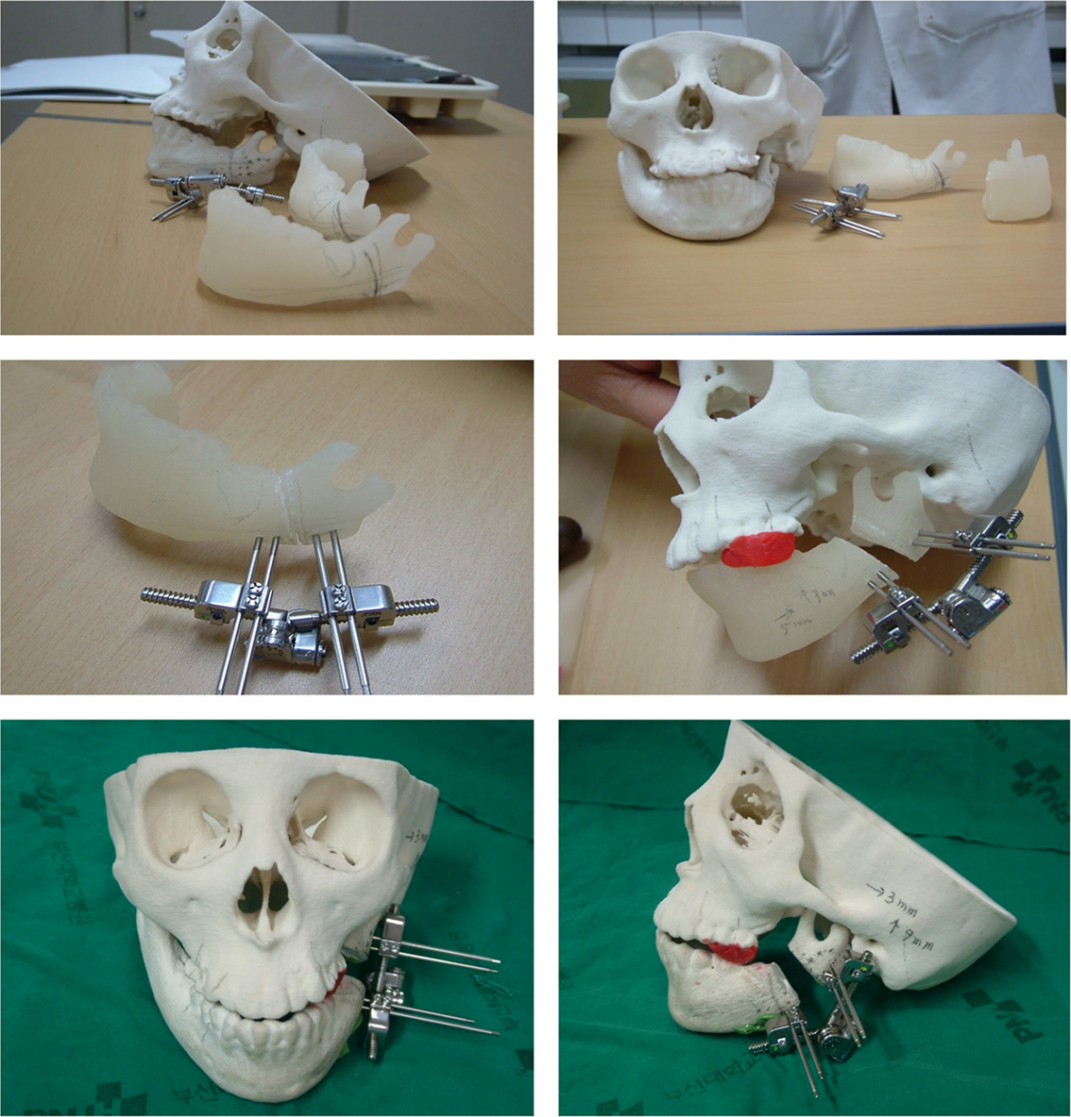
**Pre-operative model surgery with DO device.**

**Figure 3:**
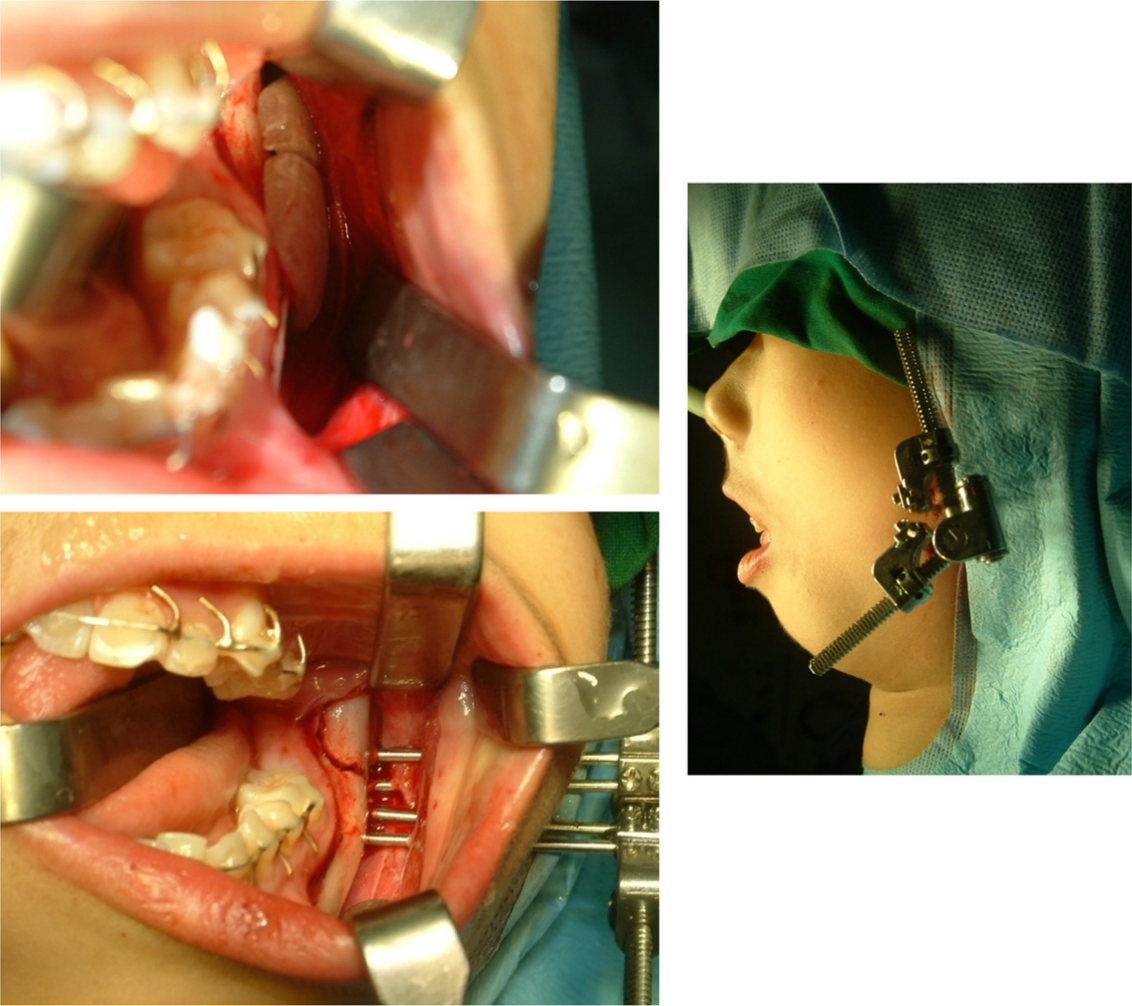
**Intra-operative photographs.**

Preoperative orthodontic treatment was administered at the age of 10 years to correct posterior openbite at the Department of Orthodontics. DO was initiated when the patient was 12 years old. The amount of horizontal and vertical DO was measured on an RP model prior to the procedure (Figure 2). From the model analysis, the required horizontal and vertical bony lengths measured were 3 mm and 16 mm, respectively. The estimated vector angle for DO was 12° from the mandibular angle. The operation included an incision on the left mandibular posterior vestibule; the lateral surface of the left mandibular body and ramus were also exposed. Furthermore, a lateral and medial corticotomy was performed at the lateral angle of the mandible, in order to create a complete greenstick fracture. An extraoral mandibular distraction device was also installed on both segments of the ramus, with the aid of screws (Leibinger, Multi-guide 2®, Switzerland) (Figure 3).

**Figure 4:**
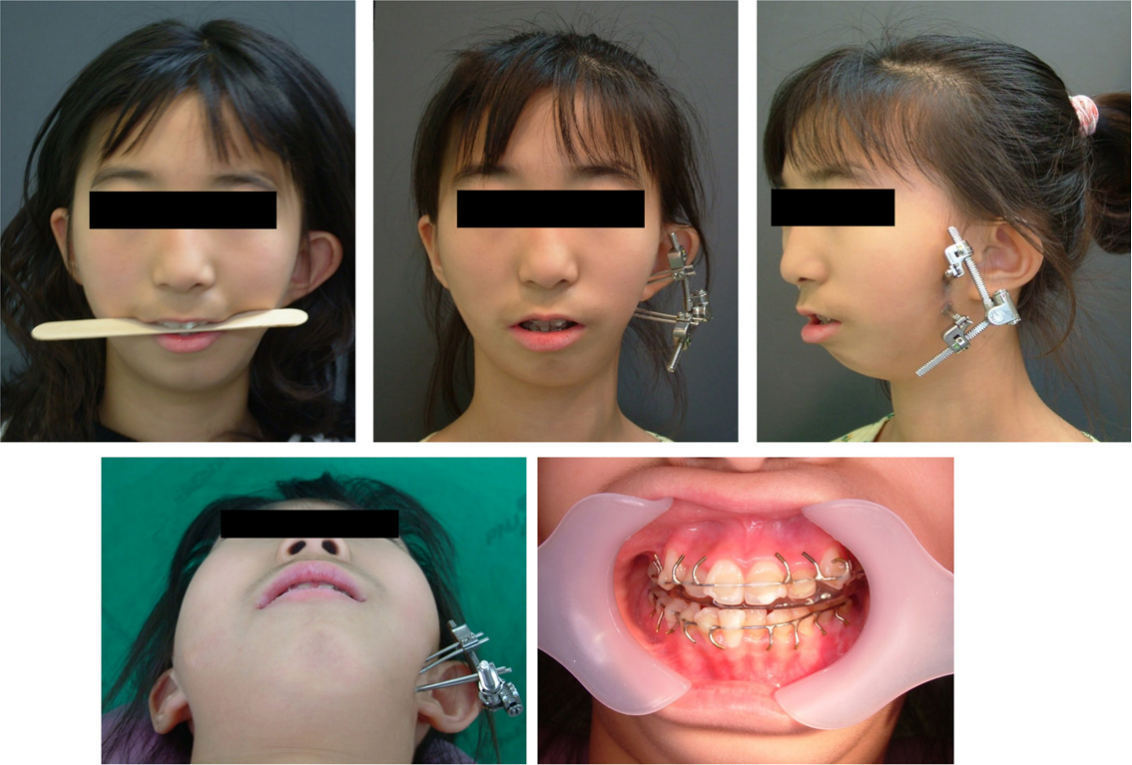
**Post-DO photographs (pre-DO, Intraoperative DO state).**

**Figure 5:**
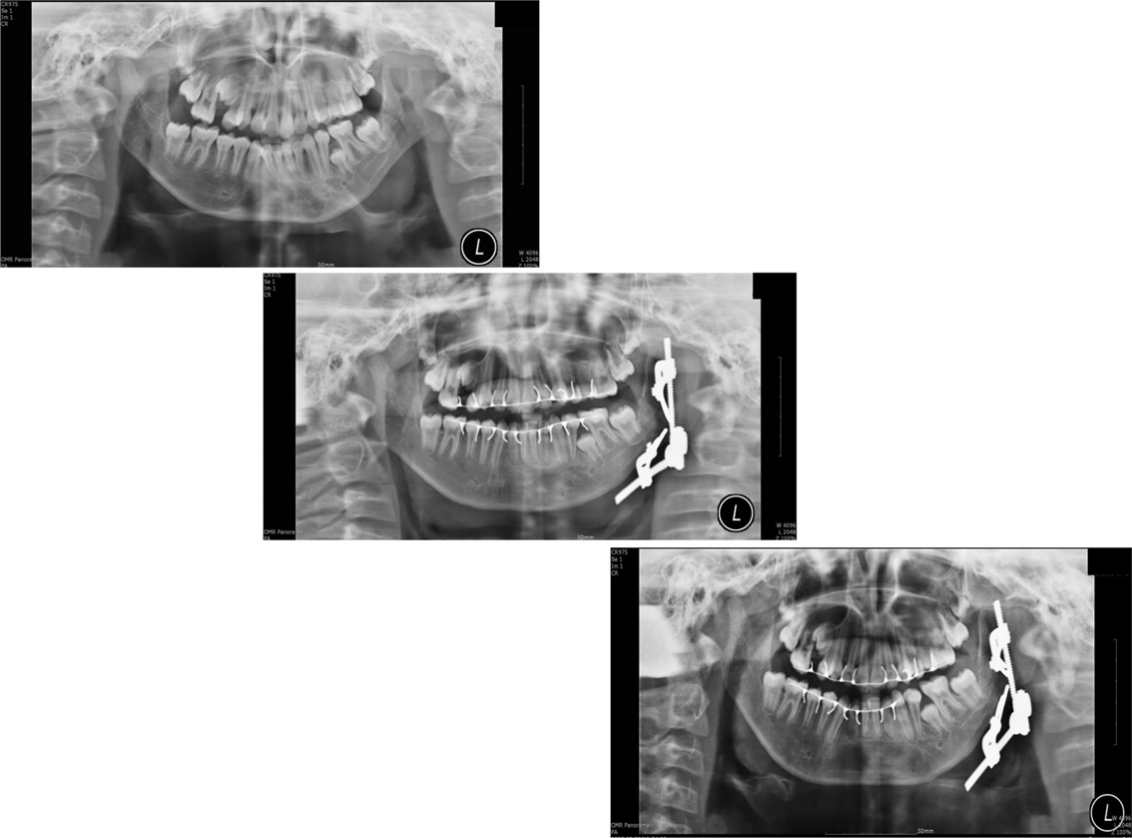
**Post-operative radiographic findings (pre-op, DO, DO with compression force).**

Seven days after the surgical procedure, DO, in an anteroposterior (A–P) direction, was initiated at a rate of 1 mm/day (two half–turns per day). Ten days after the surgery, total A–P DO (3 mm) was completed and a vertical DO, at a rate of 1 mm per a day, was resumed. The final vertical DO length was determined by multiplying the original estimated vertical DO (16 mm) by 120% in order to apply a conventional DO with stimulatory compression forces for callus molding, thereby accelerating new bone quality. Moreover, the total vertical DO length obtained was 19 mm. In the present study, DO with compression force was applied, as previously described [11,12]. After a pre-compression latency period of 3 days, compression, by reverse turning of the device, resulting in a counter force at a rate of 1 mm per a day, was initiated for 3 days. The final length of vertical DO achieved was 16 mm, similar to the original DO length (Figure 4). The distraction device was left in place for 6 weeks and was removed after the radiological evidence of mineralization was detected (Figure 5).

**Figure 6:**
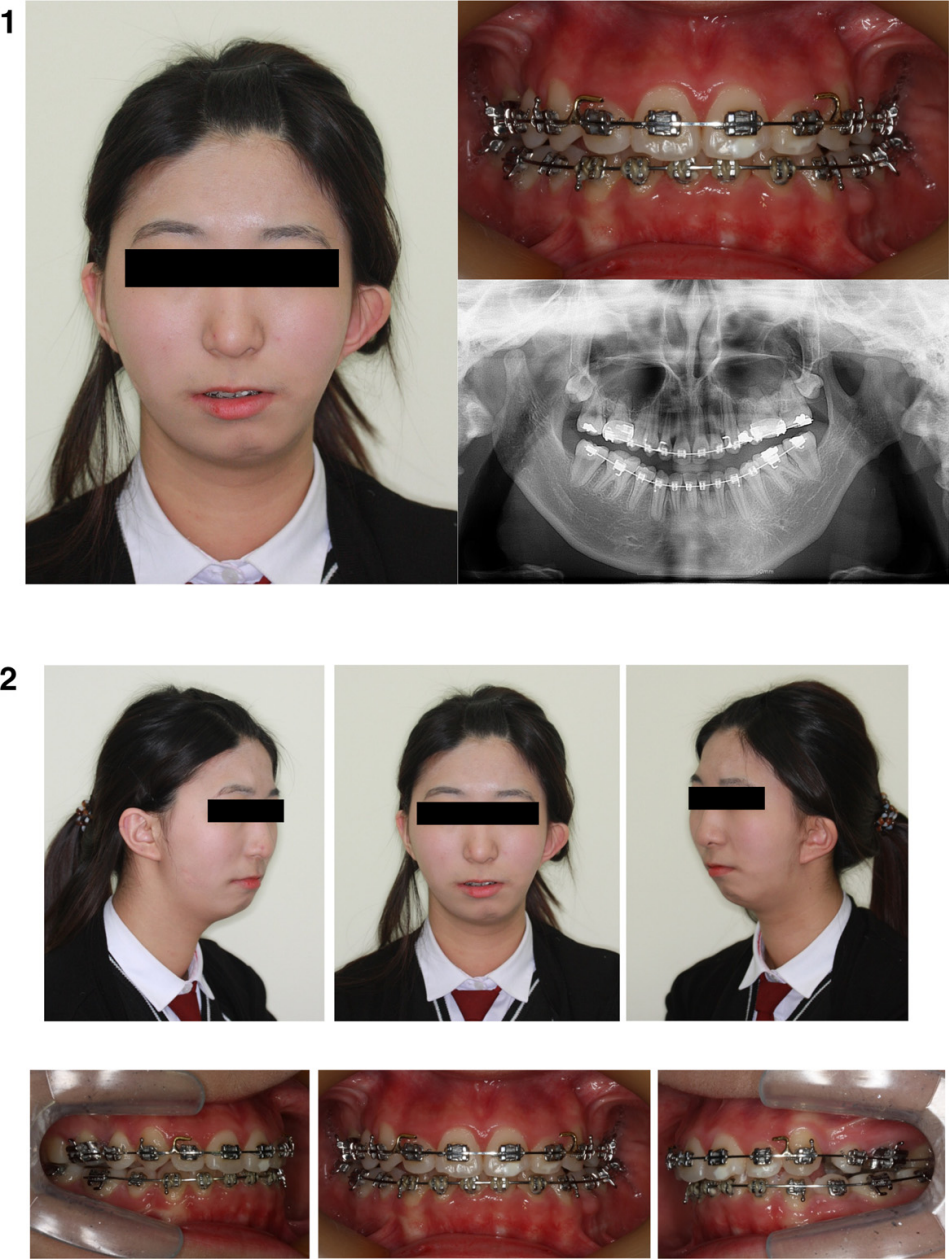
**Pre-operative view of patient.** **1.** Pre-operative photographs and panoramic view. **2.** Pre-operative extraoral and intraoral photographs.

**Figure 7:**
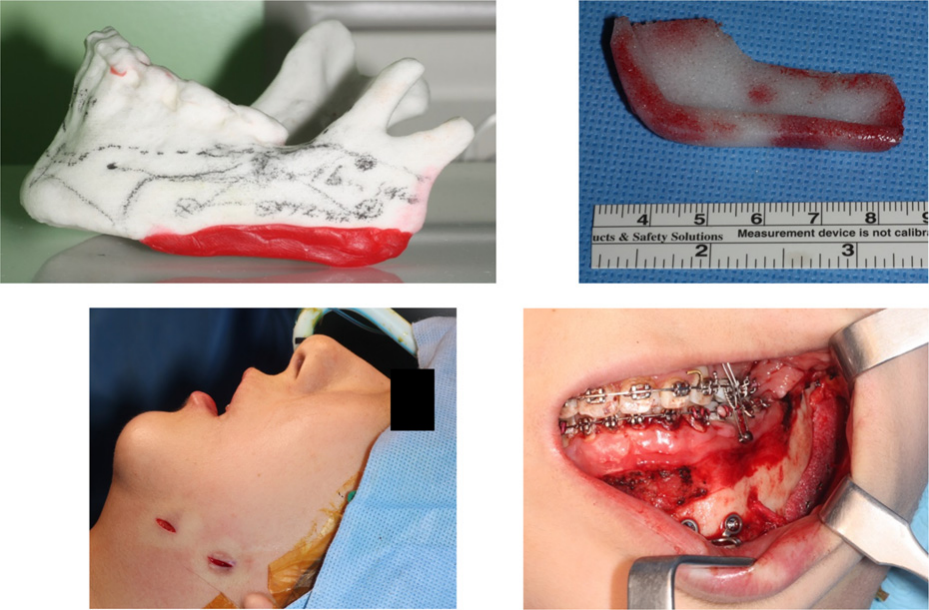
**RP model, augmentation graft and intra-operative photographs.**

**Figure 8:**
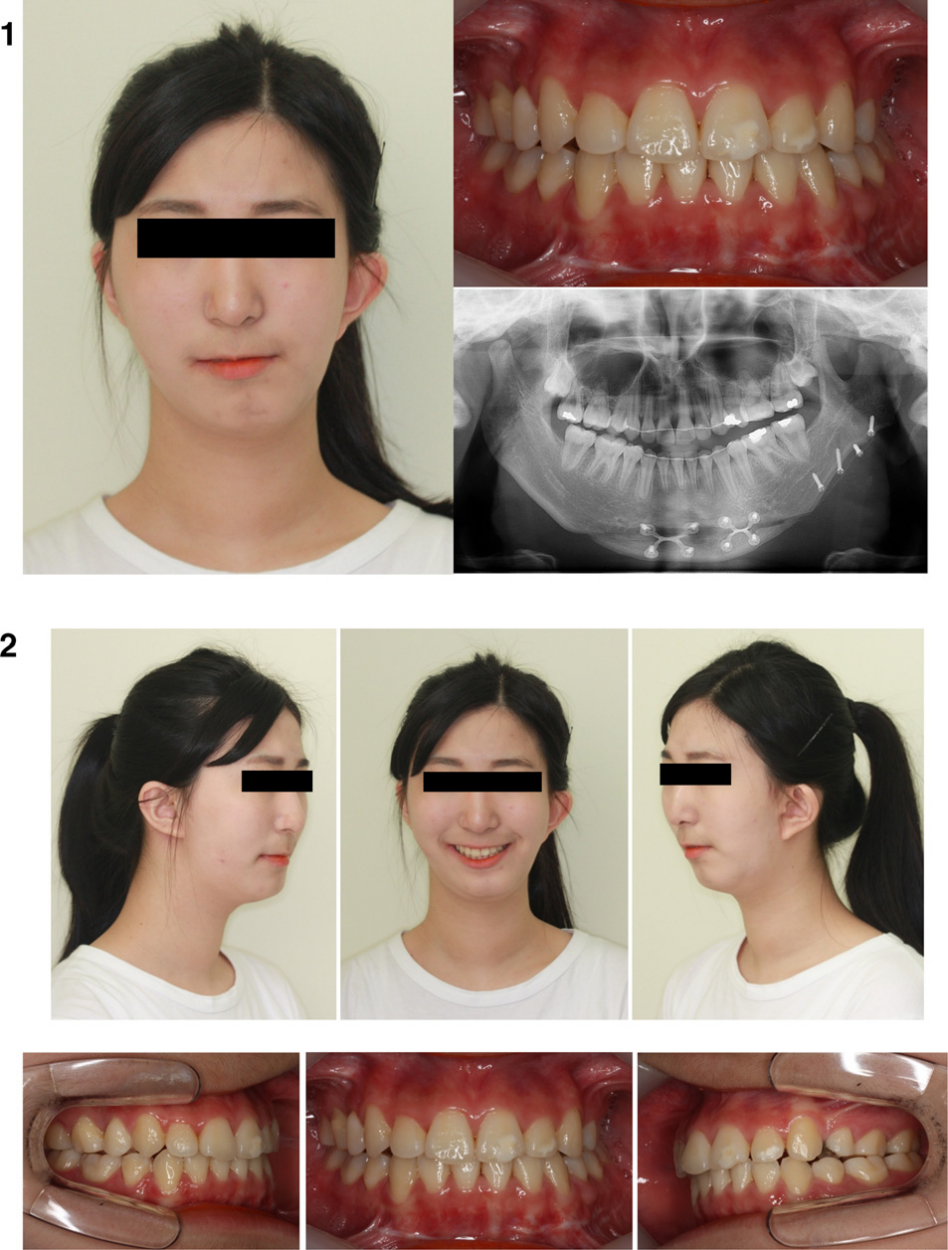
**Post-operative view of patient.** **1.** Post-operative photographs and panoramic view. **2.** Post-operative extraoral and intraoral photographs.

Subsequent to the DO procedure, orthodontic treatment was continued and periodic follow-up was conducted till the end of pubertal growth. An additional operation for the correction of facial asymmetry was performed when the patient was 18 years old. A second operation for advancement genioplasty was designed at 19 years of age, wherein the left mandibular body was augmented with a porous polyethylene implant (PPE, Medpor®, Porex) to correct the chin retrusion and mild left facial deficiency. The clinical findings of the patient at this stage were as follows: microgenia, left condyle hypoplasia, deficiency of the left mandibular body, and midline deviation of the chin to the left side (Figure 6). Advanced midline corrected genioplasty and insertion of a 5-mm thick PPE in the left ramus that was fixed with metal screws (Figure 7). Thus, the second corrective surgical procedure was completed (Figure 8).

## Discussion

The treatment goal of HFM is to attain a good facial profile without the loss of function. The severity of the temporomandibular joint (TMJ) complex deformity is the main factor influencing its reconstruction, which involves TMJ reconstruction, costochondral grafts, maxillary osteotomy, mandibular osteotomy, application of bone grafts, and distraction osteogenesis of the mandible [5,8,13–17]. Surgery, before skeletal maturity, is necessary for preventing secondary growth deformities and for cosmetic correction [17]. In general, the treatment protocol in these patients is a two-stage process, comprising DO during pubertal growth followed by a secondary orthognathic surgery at the end of pubertal growth.

DO is a technique by which a new bone is formed between the surfaces of 2 bone segments as a result of the tension that is created by the gradual movement of the two segments in the opposite direction, thereby lengthening the original bone structure [18]. This technique was first described in 1905 by Codivilla who performed osteotomies and elongated femur bones by gradual distraction [19]. It was later popularized by Ilizarov in 1951 by the elongation of the upper and lower limbs; since then, this technique has undergone several developments.

In 1973, Snyder reported mandibular lengthening by gradual distraction in animal models [20]. Mandibular lengthening by gradual distraction in a human mandible was first performed in 1992 by McCarthy, with the aid of an extraoral device in a patient with HFM [10]. Since then, it has been applied to bones of individuals with craniofacial deformities, and several studies have reported the use of this treatment, resulting in the development of an effective device.

Kim *et al.* [11] reported the effectiveness of a new DO protocol for over-distraction following compressive stimulation against the conventional DO protocol. Another study by Kim *et al.* [12] examined the expression of TGF-ß1, osteonectin, and BMP-4 in mandibular distraction osteogenesis with compression stimulation; the expression levels of TGF-ß1, osteonectin, and BMP-4 on DO with a compression force during early consolidation were increased, illustrating the effect of compression force during DO. Therefore, we applied a new DO protocol for over-distraction with compressive forces on this patient.

The DO method with compression force used in the present study is different from other conventional DO techniques because of the extended amount of distraction obtained, and the interventional as well as intermittent compression force applied during the early period of consolidation [11,12]. In the present study, after DO, vertical discrepancy had improved and the deviation of mandible was corrected to the normal midline position. The patient was treated by DO with compression force, due to which the consolidation period was shortened to 6 weeks. In general, facial skeleton growth is almost complete after the pubertal growth period. In the present study, orthognathic surgery was planned as a second stage treatment option. The patient presented with HFM type 2a, and owing to the absence of temporomandibular joint problems and the presence of mild facial asymmetry, an advanced, sliding genioplasty and left mandibular body, inferior border augmentation was planned, with the simultaneous use of a PPE implant. Mandibular body augmentation using a PPE implant is a simplified method, instead of an autograft, and provides satisfactory esthetic results by reinforcing the buccal width as well as the length of the inferior border of mandible. After the second operation, the patient’s facial profile was visibly improved. Further, periodic, close observation has been advised for this patient to assess the fate of the PPE implant. If facial asymmetry recurs, a free tissue composite flap transfer or iliac bone graft will be applied to the affected side of the face.

## Conclusion

In conclusion, satisfactory results were obtained in the patient diagnosed with HFM during the first visit. Distraction osteogenesis of the mandibular angle at an early age, followed by genioplasty and mandibular body augmentation with a PPE implant after puberty, could be sequentially performed for the final functional and esthetic reconstruction of the face.

## Consent

Written informed consent was obtained from the patient for the publication of this report and any accompanying images.
