# Erratum to: Sequential treatment for a patient with hemifacial microsomia: 10 year-long term follow up

**DOI:** 10.1186/s40902-015-0016-2

**Published:** 2015-06-17

**Authors:** Jeong-Seok Seo, Young-Chea Roh, Jae-Min Song, Won-Wook Song, Hwa-Sik Seong, Si-Yeob Kim, Dae-Seok Hwang, Uk-Kyu Kim

**Affiliations:** Department of Oral and Maxillofacial Surgery, School of Dentistry, Pusan National University, 20 Geumo-ro, Mulgeum-eup, Yangsan, 626-787 South Korea

## Erratum

After the “Competing interests” section in the original version of this article [1], an “Acknowledgements” section should be inserted and read as: **‘This research was supported by Basic Science Research Program through the National Research Foundation of Korea (NRF) funded by the Ministry of Education (2012R1A1A2003550)’.**
